# Does nutritional support contribute to mitigating the financial burden faced by TB-affected households?

**DOI:** 10.5588/ijtldopen.25.0079

**Published:** 2025-05-12

**Authors:** D. Inthavong, H. Elsayed, P. Keonakhone, V. Seevisay, S. Souksanh, S. Suthepmany, P. Siphanthong, P. Sengmany, B. Sisounon, J. Sebert, M. Yanagawa, F. Morishita, N. Nishikiori, T. Yamanaka

**Affiliations:** ^1^ National Tuberculosis Control Centre, Vientiane, Lao People’s Democratic Republic;; ^2^ Integrated Communicable Disease Control, World Health Organization Regional Office for the Western Pacific, Manila, The Philippines;; ^3^ National Nutrition Centre, Vientiane, Lao People’s Democratic Republic;; ^4^ World Health Organization Representative Office for Lao People’s Democratic Republic, Vientiane, Lao People’s Democratic Republic;; ^5^ Global Programme on Tuberculosis and Lung Health, World Health Organization, Geneva, Switzerland.

**Keywords:** tuberculosis, undernutrition, Laos, patient costs, catastrophic costs

## Abstract

**INTRODUCTION:**

Costs for nutritional supplements and food were the main driver of costs incurred by TB-affected households in Lao People’s Democratic Republic. This study assessed the impact of nutritional counselling and support on costs incurred by TB-affected households.

**METHODS:**

We conducted longitudinal data collection of costs, income, and coping mechanisms of TB-affected households within an intervention study providing nutritional counselling and support for people diagnosed with TB and having a body mass index (BMI) <18.5 kg/m^2^. Data collection tools were adapted from the WHO’s generic national TB patient cost survey questionnaire to fit a longitudinal study design. Costs were considered catastrophic when they exceeded 20% of annual household income before TB.

**RESULTS:**

A total of 268 people treated for drug-susceptible TB were included in the analysis, and the prevalence of BMI <18.5 kg/m^2^ was 38%. The intervention group had significantly lower nutritional supplement costs and direct non-medical costs after TB diagnosis than the observation group. The intervention group had less progressive catastrophic costs (+23.3 percentage points) than the observation group (+30.9 percentage points).

**CONCLUSION:**

Nutritional counselling and support were significantly associated with a reduction in the proportion of TB-affected households facing catastrophic costs due to TB.

Financial protection is a key element of universal health coverage (UHC), alongside equity, quality and availability of health services.^1,2^ It can be achieved when direct expenditures to access quality health services do not expose people to financial hardship and devastating consequences such as losing savings, taking loans or selling household assets.^3^ TB causes disability and mortality, and it remains a major public health concern in many low- and middle-income countries (LMICs).^4^ In 2023, the WHO estimated that 10.8 million people fell ill with TB globally.^5^

Despite free TB diagnostics and treatment services available in many LMICs, people with TB and their households often face substantial financial losses during an episode of TB, trapping them in a cycle of poverty.^6–8^ Recognising the financial burden of TB, the WHO’s End TB Strategy includes a target to eliminate catastrophic costs due to TB by 2020.^9^ The WHO has supported countries in implementing national TB patient cost surveys since 2015 to monitor progress toward this target. As of August 2024, 37 countries had implemented such surveys.^5^

Lao People’s Democratic Republic (PDR) is a country with a high burden of TB, with an estimated TB incidence of 132 per 100,000 in 2023.^5^ In Lao PDR, the National Tuberculosis Programme (NTP) conducted its first national TB patient cost survey in 2018–2019 using the WHO-recommended method.^10,11^ The survey found that 62.5% of households affected by TB faced catastrophic total costs,^12^ and the main cost driver was the direct non-medical costs, particularly the costs for nutritional supplements and additional food during TB treatment, which accounted for 35% of total costs.

One of the policy recommendations from the Lao PDR national survey was to improve nutritional support for people with TB through systematic nutritional assessment, counselling, and therapeutic and supplementary feeding in collaboration with the national nutrition programme of the Ministry of Health. Based on these findings, this study assessed the impact of nutritional counselling and support on mitigating catastrophic total costs due to TB in Lao PDR.

## METHODS

### Study setting

TB diagnosis and treatment are provided free of charge and covered by the NTP and the National Health Insurance (NHI) scheme. Social support equivalent to approximately USD 5 per day is provided by the NTP only for people with drug-resistant TB (DR-TB).^12^

### Study design

This intervention study aimed to assess the effect of nutritional counselling and support on TB treatment outcomes and the financial burden due to TB. The study was conducted in six central and provincial hospitals selected for their large number of TB case notifications (Supplementary Data S1). A total of 312 people with TB was the planned sample size of the study, providing 80% power to detect a minimum 12.6% reduction in the proportion of TB-affected households facing catastrophic total costs due to TB. Study participants were assigned into two groups: an observation group that received no nutritional intervention and an intervention group that received nutritional counselling and support if their body mass index (BMI) was <18.5 kg/m^2^ at TB diagnosis. For those with a BMI <18.5 kg/m^2^ at their TB diagnosis, routine nutritional counselling by a dietician and nutritional support with ready-to-use therapeutic food (RUTF) were provided until their BMI reached 18.5 kg/m^2^ during TB treatment. Further details of the interventions are available in Supplementary Data S2. The prevalence of BMI <18.5 kg/m^2^ at diagnosis and the effect of the intervention on TB treatment outcomes have been reported in detail in separate publications.^13^

### Data collection

Data collectors underwent a 5-day training programme to familiarise themselves with the study objectives, data collection tools and ethical communication practices. For enrolling study participants, they explained the purpose of this study using a printed information sheet in relevant local languages and English. People who agreed to participate and signed the informed consent form were enrolled in this study.

After TB diagnosis and obtaining informed consent, trained dieticians performed anthropometric measurements. Demographic and clinical data, and information on costs, household income and coping strategies were then collected through in-person interviews at each study site. To monitor changes in BMI and the financial burden faced by TB-affected households during treatment and anthropometric measurement, in-person interviews were conducted four times for each participant: at the start of TB treatment, at the end of the intensive phase, and the middle and end of the continuation phase. Participant enrolment and data collection commenced on 10 January 2023, and all follow-up interviews were completed by 25 January 2024.

The data collection tools for assessing costs incurred by people with TB and their households were adapted from a questionnaire from the WHO handbook for national TB household cost surveys, modified for a longitudinal study design.^10,14^ Additionally, methods from a previous longitudinal study assessing costs incurred by people with concurrent TB and diabetes and their households in the Philippines were incorporated.^15,16^

### Data analysis

Costs for each treatment phase were first interpolated using data collected from the last visit for each visit type (i.e. hospitalisation, directly observed therapy [DOT], medical follow-up and drug pick-up) based on the frequency of each visit type during each phase. Total costs for the entire TB episode were calculated by summing the costs across all phases. Costs were considered catastrophic when direct medical, direct non-medical and indirect costs exceed 20% of annual household income, as defined by the WHO (Supplementary Data S1).^10,17^

Data cleaning and processing, statistical analyses, and data visualisations were performed using R v4.4.0 (CRAN: Comprehensive R Archive Network; R Computing, Vienna, Austria). Continuous data were summarised using mean with standard deviation (SD) and 95% confidence intervals (CIs) and median with interquartile range (IQR). Categorical data were presented as frequencies with proportions. All results were stratified by the nutritional status (BMI cut-off at 18.5 kg/m^2^) at the TB diagnosis. Statistical differences were tested using the χ^2^ test for categorical data and the *t*-test (for normally distributed variables) or the Kruskal–Wallis test (for variables not normally distributed, e.g. costs, household income) for continuous data. Statistical significance was defined as *P* < 0.05. Cost and income data, collected initially in Lao Kip (LAK), were converted into United States dollars (USD) for analysis using the average UN Operational Rates of Exchange during the data collection period at the rate of LAK19,150 to USD1.

Univariate logistic regression analysis was conducted to identify variables associated with catastrophic total costs due to TB. Multivariate backward stepwise logistic regression was performed to identify the best model based on the Akaike Information Criterion. The selected final model was used for multivariate logistic regression analysis to calculate the adjusted odds ratio (AOR).

### Ethical considerations

A written consent form was obtained from each participant before enrolment, explicitly stating that only the principal investigator (PI) and co-PIs could access the study dataset. Before obtaining written informed consent, data collectors explained the purpose of this research with a written information sheet at each study site. Participant’s voluntary will to continue participating in this study was asked and confirmed also at each data collection. Ethics approvals were also obtained from the Lao PDR National Ethics Committee for Health Research, Vientiane, Lao (Ref: 021/NECHR) and the Ethics Review Committee of the WHO Regional Office for the Western Pacific, Manila, The Philippines (Ref: 2022.3.LAO.1.ETB).

## RESULTS

### Demographic and clinical characteristics

A total of 297 people treated for drug-susceptible TB (DS-TB) were enrolled, and of them, 268 completed all four times of the data collection (Supplementary Figure S3 and Supplementary Table S4), with a mean age of 47.8 years. The overall prevalence of BMI <18.5 kg/m^2^ was 37.7% (Table 1). The majority (94.8%) were newly diagnosed TB cases, and 11.9% were people living with HIV. Comparison between the observation and intervention groups revealed no significant differences in most demographic and clinical characteristics, except for the status of the primary income earner: 24.5% in the observation group vs 44.2% in the intervention group (*P* = 0.001) (Table 1).

**Table 1.** Socio-economic status of study participants who completed four data collections.

Variables	Observation group	Intervention group	All participants	
	*n* (%)	*n* (%)	*n* (%)	*P*-value
Total	139 (51.9)	129 (48.1)	268 (100)	
Demographic characteristics				
Age group, years				
0–14	1 (0.7)	0 (0.0)	1 (0.4)	0.373
15–24	14 (10.1)	10 (7.8)	24 (9.0)	
25–34	27 (19.4)	23 (17.8)	50 (18.7)	
35–44	15 (10.8)	22 (17.1)	37 (13.8)	
45–54	25 (18.0)	26 (20.2)	51 (19.0)	
55–64	24 (17.3)	28 (21.7)	52 (19.4)	
≥65	33 (23.7)	20 (15.5)	53 (19.8)	
Sex				
Female	62 (44.6)	44 (34.1)	106 (39.6)	0.103
Male	77 (55.4)	85 (65.9)	162 (60.4)	
Education level				
No education	18 (12.9)	8 (6.2)	26 (9.7)	0.251
Primary	36 (25.9)	34 (26.4)	70 (26.1)	
Lower/higher secondary	62 (44.6)	59 (45.7)	121 (45.1)	
Diploma or higher, vocational, other	23 (16.5)	28 (21.7)	51 (19.0)	
Insurance status				
None	46 (33.1)	49 (38.0)	95 (35.4)	0.393
National Health Insurance (NHI) scheme	78 (56.1)	70 (54.3)	148 (55.2)	
Community-Based Health Insurance (CBHI)	2 (1.4)	0 (0.0)	2 (0.7)	
Health Equity Fund (HEF)	0 (0.0)	0 (0.0)	0 (0.0)	
Social Security Organization (SSO) for salaried private sector employees	5 (3.6)	4 (3.1)	9 (3.4)	
State Authority for Social Security (SASS) for civil servants	5 (3.6)	5 (3.9)	10 (3.7)	
Private health insurance	3 (2.2)	0 (0.0)	3 (1.1)	
Other	0 (0.0)	1 (0.8)	1 (0.4)	
Employment status before TB				
Unemployed	38 (27.3)	29 (22.5)	67 (25.0)	0.076
Formal paid work	18 (12.9)	22 (17.1)	40 (14.9)	
Informal paid work	60 (43.2)	68 (52.7)	128 (47.8)	
Retired/student/housework/other	23 (16.5)	10 (7.8)	33 (12.3)	
Household size				
≥5	67 (48.2)	63 (48.8)	130 (48.5)	1.000
<5	72 (51.8)	66 (51.2)	138 (51.5)	
Primary income earner				
No	99 (71.2)	63 (48.8)	162 (60.4)	0.001
Yes	34 (24.5)	57 (44.2)	91 (34.0)	
Equal contributor	6 (4.3)	9 (7.0)	15 (5.6)	
Clinical characteristics				
TB type				
Pulmonary, bacteriologically confirmed	99 (71.2)	90 (69.8)	189 (70.5)	0.787
Pulmonary, bacteriologically unconfirmed	34 (24.5)	31 (24.0)	65 (24.3)	
Extrapulmonary	6 (4.3)	8 (6.2)	14 (5.2)	
Treatment history				
New	131 (94.2)	123 (95.3)	254 (94.8)	0.390
Relapse	6 (4.3)	6 (4.7)	12 (4.5)	
Retreatment (other than relapse)	2 (1.4)	0 (0.0)	2 (0.7)	
HIV status				
HIV-positive	16 (11.5)	16 (12.4)	32 (11.9)	0.565
HIV-negative	123 (88.5)	112 (86.8)	235 (87.7)	
Status unknown	0 (0.0)	1 (0.8)	1 (0.4)	
Diagnostic delay* (>4 weeks)	74 (53.2)	59 (45.7)	133 (49.6)	0.269
Body mass index at TB diagnosis, kg/m^2^				
<18.5	57 (41.0)	44 (34.1)	101 (37.7)	0.299
≥18.5	82 (59.0)	85 (65.9)	167 (62.3)	
Hospitalised due to TB^†^				
Until TB diagnosis	39 (28.1)	32 (24.8)	71 (26.5)	0.643
In intensive phase	8 (5.8)	4 (3.1)	12 (4.5)	0.451
Until middle of continuation phase	1 (0.7)	0 (0.0)	1 (0.4)	1.000
Financial status	Mean (95% CI)	Mean (95% CI)	Mean (95% CI)	
Self-reported monthly household income, USD				
Before onset of TB symptoms	336 (256–416)	398 (283–512)	366 (297–434)	0.376
At the time of TB diagnosis	279 (206–352)	358 (244–473)	317 (251–384)	0.243
At the end of intensive phase	275 (208–342)	345 (254–436)	309 (253–364)	0.213
At the middle of continuation phase	348 (238–458)	352 (252–453)	350 (276–424)	0.956
At the end of continuation phase	305 (235–374)	353 (264–441)	328 (272–383)	0.399
Social welfare payment	*n* ()	*n* ()	*n* ()	
Before TB diagnosis	1 (0.7)	—	1 (0.4)	1.000
Intensive phase	4 (2.9)	—	4 (1.5)	0.151
Middle of continuation phase	—	—	—	—
End of continuation phase	—	—	—	—
Vouchers				
Before TB diagnosis	3 (2.2)	—	3 (1.1)	0.273
Intensive phase	—	—	—	—
Middle of continuation phase	—	—	—	—
End of continuation phase	—	—	—	—
Health service utilisation (times of facility visits)	Mean (95% CI)	Mean (95% CI)	Mean (95% CI)	
Before TB diagnosis				
Care seeking	2.5 (2.2–2.8)	2.0 (1.8–2.2)	2.3 (2.1–2.5)	0.006
Intensive phase				
Medical follow-up	0.8 (0.7–0.9)	0.9 (0.8–0.9)	0.8 (0.8–0.9)	0.224
Drug pick-up	4.6 (3.5–5.8)	4.4 (4–4.8)	4.5 (3.9–5.1)	0.671
Directly observed therapy	0.4 (0–0.7)	—	0.2 (0–0.4)	0.033
Middle of continuation phase				
Medical follow-up	0.6 (0.5–0.6)	0.6 (0.5–0.6)	0.6 (0.5–0.6)	0.945
Drug pick-up	3.2 (2.8–3.6)	3.2 (2.8–3.7)	3.2 (2.9–3.5)	0.877
Directly observed therapy	0.1 (0–0.2)	—	— (0–0.1)	0.319
End of continuation phase				
Medical follow-up	0.8 (0.7–0.9)	0.9 (0.8–1)	0.8 (0.8–0.9)	0.419
Drug pick-up	3.4 (3–3.9)	3.3 (2.9–3.8)	3.4 (3.1–3.7)	0.729
Directly observed therapy	0.1 (0–0.2)	—	—	0.319
Total (before TB diagnosis until end of continuation phase)	16.4 (14.8–18.1)	15.2 (14.0–16.4)	15.9 (14.8–16.9)	0.240

* >4 weeks from onset of TB symptoms.

† No hospitalisations were reported between the middle and end of the TB continuation phase.

CI = confidence interval.

### Changes in reported monthly household income

The mean reported monthly household income before having TB was USD366 (95% CI 297–434), with no significant differences between the two groups. The reported monthly household income declined at TB diagnosis (USD317, 95% CI 251–384) and further at the end of the intensive phase of TB treatment (USD309, 95% CI 253–364) (Table 1), but showed partial recovery by the middle (USD350, 95% CI 276–424) and end of the continuation phase (USD328, 95% CI 272–393). No significant differences between the two groups were observed in the household income during TB treatment. Social welfare support, such as paid sick leave, benefits and cash transfers, was minimal throughout the TB episodes, with only 1.5% of participants receiving assistance during the intensive phase (Table 1). A high incidence of job loss was observed before TB diagnosis (19.8%) and during the intensive phase (11.9%), which may have resulted in dissaving and taking loans to cope with the financial burden due to TB during these periods (Supplementary Figure S5).

### Health service utilisation

The mean total number of health facility visits was 15.9 per participant. Of these, 2.3 times were before TB diagnosis, 5.5 times during the intensive phase, 4.8 times until the middle of the continuation phase, and 4.2 times until the end of the continuation phase (Table 1). Drug pick-up accounted for the highest number of visits (11.1 times), followed by care-seeking (2.3 times) and medical follow-up (2.2 times). Overall, 26.5% of participants were hospitalised due to TB at some point during their care-seeking, and the proportion dropped during TB treatment (intensive phase: 4.5%, middle of continuation phase: 0.4%). Significant differences between the two groups were observed in the mean number of care-seeking visits (observation group: 2.5 times, intervention group: 2.0 times, *P* = 0.006) and DOT visits during the intensive phase (observation group: 0.4 times, intervention group: 0 times, *P* = 0.033) (Table 1).

### Costs incurred by TB-affected households

Overall, the mean total cost was USD780 (Table 2), with income loss accounting for the largest share (64.8%), followed by direct non-medical costs (27.2%) and direct medical costs (8.0%). While total costs did not differ significantly between the two groups, participants in the intervention group reported significantly lower nutritional supplement costs (USD108 vs USD169; *P* = 0.01) and direct non-medical costs (USD119 vs USD181; *P* = 0.008) after TB diagnosis (Table 2). This resulted in significantly lower total direct non-medical costs in the intervention group (USD169 vs USD251; *P* = 0.004).

**Table 2.** Detail of costs incurred per TB-affected households.

Costs incurred by TB-affected households, US$	Observation group		Intervention group		All participants		*P*-value
	Mean (95%CI)	%	Mean (95%CI)	%	Mean (95%CI)	%	
Pre-TB diagnosis							
Direct medical costs	61.91 (43.74–80.07)	7.4	53.87 (34.57–73.18)	7.6	58.04 (44.88–71.2)	7.4	0.549
Direct non-medical costs							
Other non-medical costs	25.62 (19.29–31.95)	3.0	18.59 (9.92–27.26)	2.6	22.23 (16.94–27.53)	2.9	0.192
Nutrition supplement	44.64 (29.24–60.03)	5.3	31.54 (15.4–47.68)	4.4	38.33 (27.23–49.43)	4.9	0.246
Total	70.25 (53.1–87.41)	8.3	50.13 (31.6–68.66)	7.0	60.57 (47.99–73.14)	7.8	0.116
Income loss	153.74 (8.04–299.43)	18.3	69.19 (18.64–119.75)	9.7	113.04 (33.97–192.11)	14.5	0.294
Post-TB diagnosis							
Direct medical costs							
Drug pick-up	0.08 (0–0.25)	0.0	0 (-)	0.0	0.04 (0–0.13)	0.0	—
Directly observed therapy	- (-)	0.0	0 (-)	0.0	- (-)	0.0	0.364
Follow-up	1.62 (0.99–2.25)	0.2	1.13 (0.25–2)	0.2	1.38 (0.85–1.91)	0.2	0.107
Hospitalisation	5.50 (0.13–10.87)	0.7	0.79 (0–2.28)	0.1	3.24 (0.36–6.11)	0.4	0.083
Total	7.21 (1.64–12.77)	0.9	1.92 (0.22–3.63)	0.3	4.66 (1.66–7.66)	0.6	0.126
Direct non-medical costs							
Drug pick-up	0.02 (0–0.05)	0.0	0.52 (0–1.19)	0.1	0.26 (0–0.58)	0.0	—
Directly observed therapy	0.67 (0–1.4)	0.1	0 (-)	0.0	0.35 (0–0.73)	0.0	0.078
Follow-up	8.49 (6.5–10.49)	1.0	10.34 (5.99–14.7)	1.4	9.38 (7.06–11.71)	1.2	0.435
Hospitalisation	2.96 (0.26–5.66)	0.4	0.43 (0–1.05)	0.1	1.74 (0.31–3.17)	0.2	0.082
Nutrition supplement	168.62 (133.36–203.87)	20.0	107.9 (78.63–137.18)	15.1	139.39 (116.17–162.62)	17.9	0.010
Total	180.76 (145.81–215.71)	21.5	119.19 (90.25–148.14)	16.7	151.13 (128.11–174.15)	19.4	0.008
Incomeloss	367.90 (192.78–543.01)	43.7	418.86 (204.39–633.32)	58.7	392.43 (255.79–529.06)	50.3	0.714
Total direct medical costs	69.11 (49.72–88.5)	8.2%	55.8 (36.45–75.15)	7.8	62.70 (49.06–76.34)	8.0	0.338
Total direct non-medical costs	251.01 (210.08–291.95)	29.8%	169.32 (131.99–206.65)	23.7	211.69 (183.62–239.76)	27.2	0.004
Total income loss	521.63 (211.56–831.71)	62.0%	488.05 (247.30–728.81)	68.4	505.47 (308.49–702.45)	64.8	0.867
Total cost	841.76 (522.86–1160.66)	100.0	713.17 (460.91–965.43)	100.0	779.86 (575.83–983.90)	100.0	0.536

CI = confidence interval.

### Catastrophic total costs due to TB and its associated risk factors

**Figure 1.**
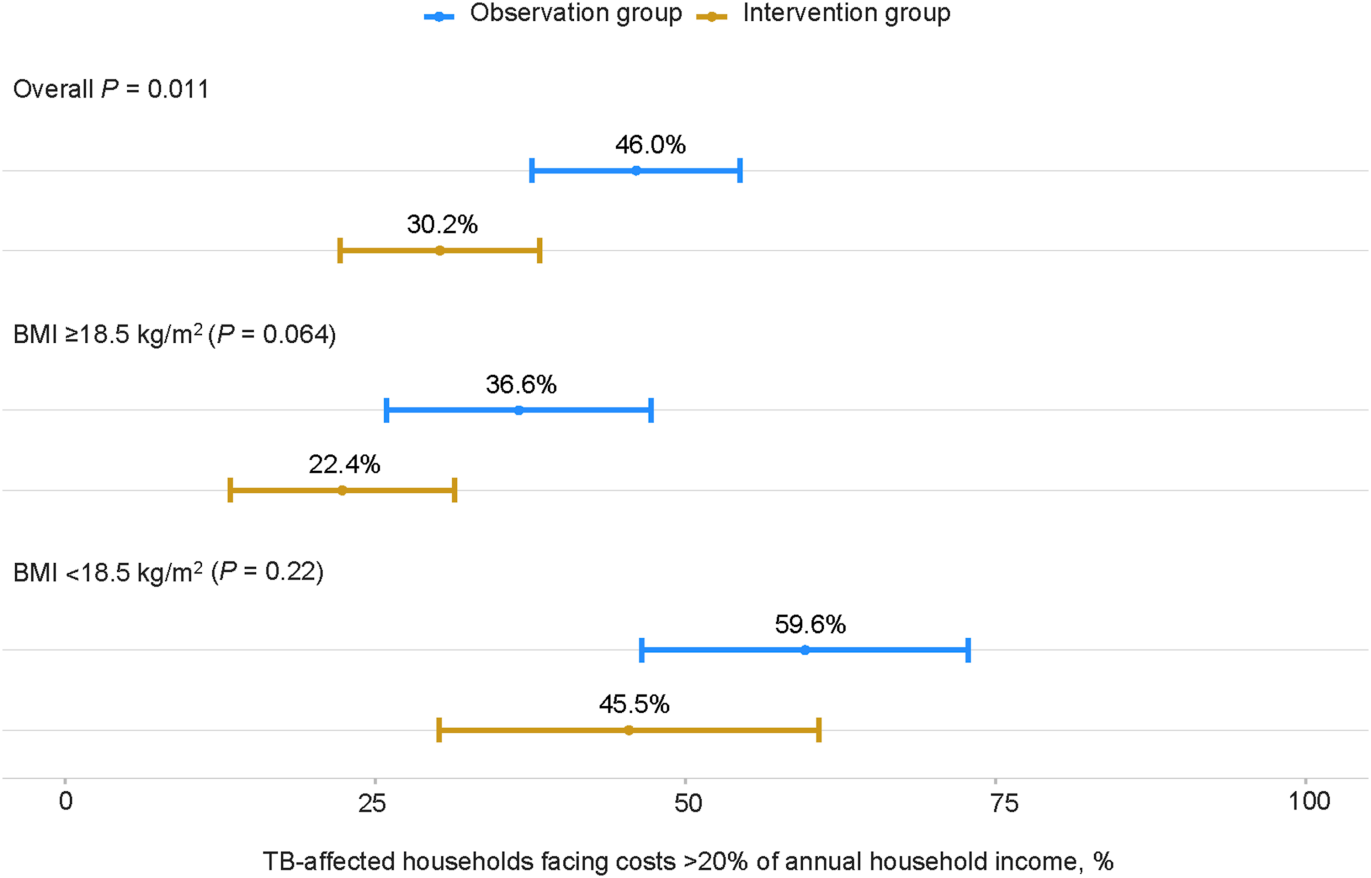
Percentage of TB-affected households facing costs >20% of annual household income by study group and body mass index at TB diagnosis.

**Figure 2.**
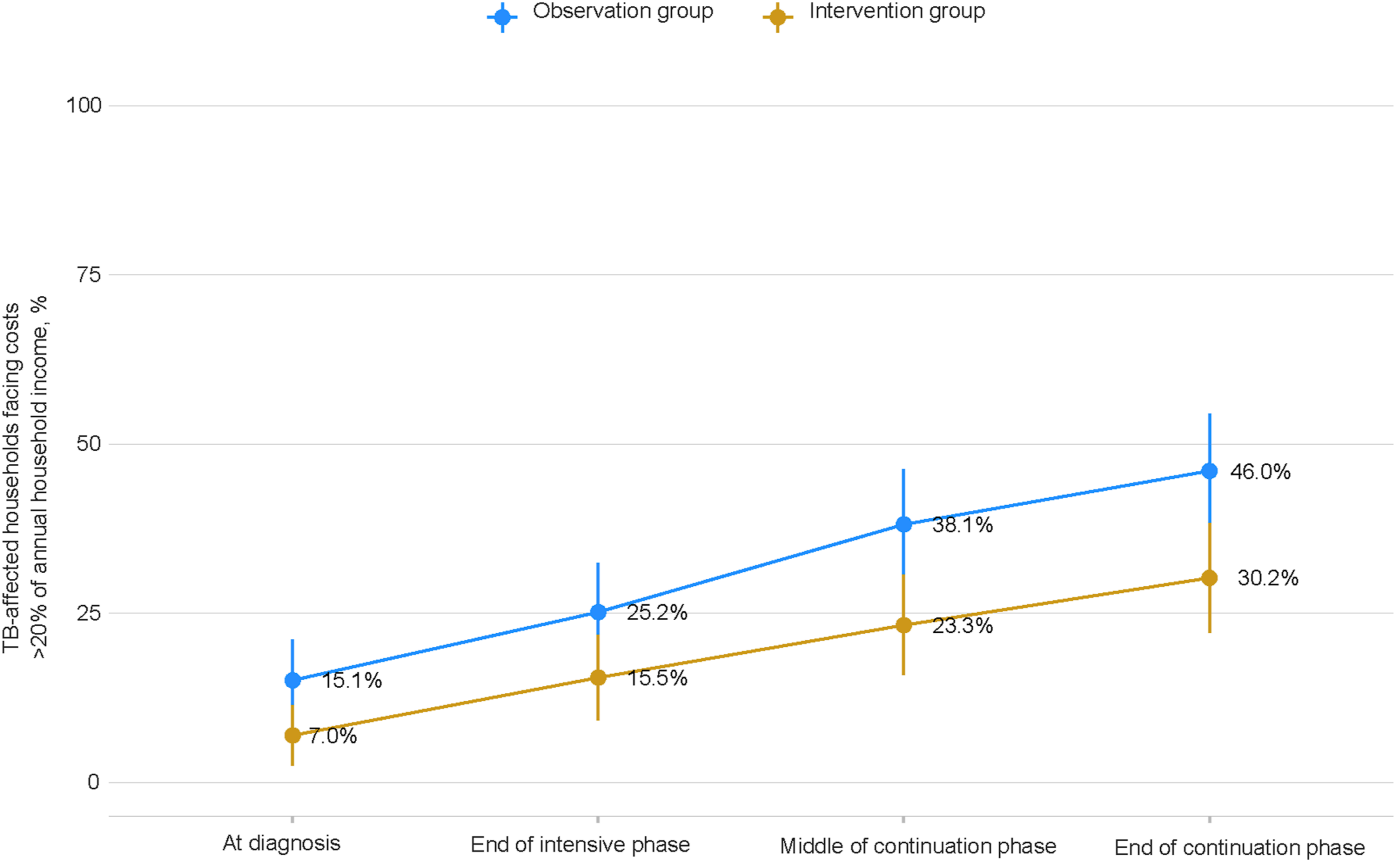
Percentage of TB-affected households facing costs >20% of annual household income by TB treatment phase.

The proportion of TB-affected households facing catastrophic total costs was significantly lower in the intervention group (30.2%, 95% CI 22.2–38.3) compared to the observation group (46.0%, 95% CI 37.7–54.4) with a *P*-value of 0.011 (Figure 1), while at the TB diagnosis, the proportion was lower in the intervention group (7.0%) compared to the observation group (15.1%). In both groups, the proportion was higher among people with BMI <18.5 kg/m^2^ at TB diagnosis than those with BMI ≥18.5 kg/m^2^ (Figure 1). The incidence of catastrophic total costs increased progressively throughout the TB episode. A lower incremental incidence was observed during TB treatment in the intervention group (+23.3 per cent points) compared to the observation group (+30.9 per cent points) (Figure 2).

Multivariate logistic regression identified providing nutritional counselling and support as a significant protective factor against the incidence of catastrophic total costs with an AOR of 0.49 (95% CI 0.28–0.87; *P* = 0.015). Other significant protective factors included BMI ≥18.5 kg/m^2^ at TB diagnosis (AOR 0.31; *P* < 0.001), higher income quintiles (e.g. fifth quintile: AOR 0.11; *P* < 0.001), early diagnosis (AOR 0.48; *P* = 0.012) (Table 3).

**Table 3.** Factors associated with facing costs >20% of annual household income.

Variable	*N*	*n* (%)	*P* value	Univariable		Multivariable	
				cOR (95% CI)	*P* value	aOR (95%CI)	*P* value
Age group, years							
65+	53	20 (37.7)	0.935	Reference			
0–44	112	42 (37.5)		0.99 (0.51–1.96)	0.977	—	
45–64	103	41 (39.8)		1.09 (0.55–2.18)	0.802	—	
Sex							
Male	162	58 (35.8)	0.334	Reference			
Female	106	45 (42.5)		1.32 (0.80–2.19)	0.274	—	
Marital status							
Single	66	21 (31.8)	0.325	Reference		—	
Married	163	64 (39.3)		1.39 (0.76–2.57)	0.292	—	
Divorced/separated/widowed	39	18 (46.2)		1.84 (0.81–4.18)	0.144	—	
Insurance status							
No insurance	95	30 (31.6)	0.115	Reference			
With insurance	173	73 (42.2)		1.58 (0.94–2.70)	0.088	—	
Smoking history							
Current smoker	23	8 (34.8)	0.915	Reference			
No smoking experience	140	55 (39.3)		1.21 (0.49–3.19)	0.681	—	
Ex-smoker	105	40 (38.1)		1.15 (0.46–3.09)	0.766	—	
Alcohol use							
Daily	23	7 (30.4)	0.266	Reference			
Weekly	26	6 (23.1)		0.69 (0.19–2.46)	0.561	—	
Monthly	26	10 (38.5)		1.43 (0.44–4.84)	0.556	—	
Rarely/Never	193	80 (41.5)		1.62 (0.66–4.38)	0.312	—	
Education level							
No education	26	14 (53.8)	<0.001	Reference			
Primary	70	38 (54.3)		1.02 (0.41–2.52)	0.969	—	
Lower/higher secondary	121	41 (33.9)		0.44 (0.18–1.04)	0.060	—	
Diploma or higher, vocational, other	51	10 (19.6)		0.21 (0.07–0.58)	0.003	—	
Employment status before TB							
Unemployed	67	32 (47.8)	0.056	Reference			
Formal paid work	40	11 (27.5)		0.41 (0.17–0.95)	0.041	1.14 (0.42–3.06)	0.789
Informal paid work	128	52 (40.6)		0.75 (0.41–1.36)	0.340	1.28 (0.65–2.59)	0.481
Retired/student/housework/other	33	8 (24.2)		0.35 (0.13–0.86)	0.027	0.30 (0.10–0.84)	0.026
Primary income earner							
No	162	63 (38.9)	0.964	Reference			
Yes	91	34 (37.4)		0.94 (0.55–1.59)	0.811	—	
Equal contributor	15	6 (40.0)		1.05 (0.34–3.05)	0.933	—	
TB type							
Pulmonary, bacteriologically confirmed	189	66 (34.9)	0.178	Reference			
Pulmonary, bacteriologically unconfirmed	65	31 (47.7)		1.70 (0.96–3.01)	0.069	—	
Extrapulmonary	14	6 (42.9)		1.40 (0.44–4.19)	0.551	—	
TB history							
Relapse/retreatment	14	6 (42.9)	0.946	Reference			
New	254	97 (38.2)		0.82 (0.28–2.57)	0.727	—	
HIV status							
HIV positive	32	13 (40.6)	0.428	Reference			
HIV negative	235	89 (37.9)		0.89 (0.42–1.93)	0.764	—	
Status unknown	1	1 (100.0)		3095801.51 (0.00–NA)	0.986	—	
Household size							
≥5	130	43 (33.1)	0.104	Reference			
<5	138	60 (43.5)		1.56 (0.95–2.57)	0.081	—	
Diagnostic delay							
Yes	133	62 (46.6)	0.009	Reference			
No	135	41 (30.4)		0.50 (0.30–0.82)	0.007	0.48 (0.27–0.85)	0.012
Body mass index at TB diagnosis							
<18.5 kgm^2^	101	54 (53.5)	<0.001	Reference			
≥18.5 kg/m^2^	167	49 (29.3)		0.36 (0.22–0.60)	0.001	0.31 (0.17–0.55)	0.001
Nutritional intervention							
Observation group	139	64 (46.0)	0.011	Reference			
Intervention group	129	39 (30.2)		0.51 (0.31–0.84)	0.008	0.49 (0.28–0.87)	0.015
Household income quintile before TB							
First (lowest)	45	31 (68.9)	<0.001	Reference			
Second	56	21 (37.5)		0.27 (0.12–0.61)	0.002	0.23 (0.09–0.57)	0.002
Third	41	16 (39.0)		0.29 (0.12–0.69)	0.006	0.34 (0.13–0.87)	0.028
Fourth	72	23 (31.9)		0.21 (0.09–0.46)	0.001	0.20 (0.08–0.49)	0.001
Fifth (highest)	54	12 (22.2)		0.13 (0.05–0.31)	0.001	0.11 (0.04–0.29)	0.001

* >4 weeks from onset of TB symptoms.

cOR = crude odds ratio; CI = confidence interval; aOR = adjusted odds ratio; NA = not available.

## DISCUSSION

This study assessed the impact of nutritional counselling and support on averting catastrophic total costs due to TB. The study provided evidence that the nutritional counselling and support provided as an intervention in this study, along with other factors such as BMI at TB diagnosis, household income before TB and early diagnosis, were significantly associated with a lower proportion of TB-affected households facing catastrophic total costs due to TB (intervention group: 30.2%, observation group: 46.0%; *P* = 0.011), and the interventions were associated with lower nutritional supplement costs (intervention group: USD108, observation group: USD169; *P* = 0.01) and total direct non-medical costs (intervention group: USD119, observation group: USD181; *P* = 0.008).

The results of this study support the need for a national policy in Lao PDR to integrate the nutritional assessment at TB diagnosis and ensure consecutive nutritional counselling and support throughout treatment for all people with TB. Given that the people with BMI <18.5 kg/m^2^ were more likely to face catastrophic total costs due to TB and that undernutrition is more prevalent in rural areas in Lao PDR,^18,19^ a nationwide intervention rather than a selective approach would be essential for addressing undernutrition and financial burden among TB-affected households. Scaling up such an initiative will require strategic discussions on funding allocation and the integration of a monitoring mechanism of the nutritional status of people with TB into routine TB surveillance systems. This study also found that early TB diagnosis was associated with a lower proportion of TB-affected households facing catastrophic total costs, which was consistent with previous evidence that showed active TB case finding was associated with lower pre-treatment and total costs in Cambodia and Viet Nam.^20,21^ Expanding access to rapid TB diagnostic services and active case-finding efforts would also contribute to averting catastrophic total costs and improving financial protection for TB-affected households.^22^ Also, our finding that income loss accounted for 65% of total costs highlighted an action required to enhance social protection policy, e.g., job protection and cash transfer mechanisms, for people affected by TB to mitigate the catastrophic total costs due to TB in Lao PDR. NTP may need to explore a collaboration with relevant government bodies/stakeholders for social protection policies, referencing policy actions taken in other countries, e.g. the Philippines and Viet Nam.^23,24^

Our study has several limitations. First, the results were not nationally representative, as the study was conducted in selected central and provincial hospitals, excluding health facilities in rural areas where people may face higher healthcare costs. Compared to the 2018–2019 national TB household cost survey, this study reported a lower proportion of TB-affected households experiencing catastrophic total costs and a lower contribution of direct non-medical costs. However, these differences should not be interpreted as progress toward the global and national target of no TB-affected households facing catastrophic total costs due to TB. A second national TB household cost survey will be required to provide a more comprehensive picture. Second, this study did not include people with DR-TB due to the limited number of cases in the country. Given that people with DR-TB are more likely to be with undernutrition, future policies that address TB-related undernutrition should also cover those with DR-TB.^13,25^

## CONCLUSION

This study was the first to assess the impact of nutritional counselling and support on averting catastrophic total costs due to TB. The findings suggest that nutritional interventions could help mitigate the financial burden of TB-affected households by reducing direct non-medical costs. These findings could be translated into national policies to scale up the nutritional interventions for people with TB in Lao PDR. Further investigations are required to assess the generalisability of the study results in other country settings and the cost-effectiveness of the intervention.

## Supplementary Material



## References

[1] Boerma T, Monitoring progress towards universal health coverage at country and global levels. PLoS Med. 2014;11(9):e1001731. 25243899 10.1371/journal.pmed.1001731 PMC4171369

[2] McIntyre D, Promoting universal financial protection: evidence from seven low- and middle-income countries on factors facilitating or hindering progress. Health Res Policy Syst. 2013;11:36. 24228762 10.1186/1478-4505-11-36 PMC3848816

[3] World Health Organization, World Bank. Tracking universal health coverage: 2021 global monitoring report. Geneva, Switzerland: WHO, 2022.

[4] Portnoy A, Costs incurred by people receiving tuberculosis treatment in low-income and middle-income countries: a meta-regression analysis. Lancet Glob Health. 2023;11(10):e1640–e1647. 37734806 10.1016/S2214-109X(23)00369-8 PMC10522775

[5] World Health Organization. Global tuberculosis report, 2024. Geneva, Switzerland: WHO, 2024.

[6] Tanimura T, Financial burden for tuberculosis patients in low- and middle-income countries: a systematic review. Eur Respir J. 2014;43(6):1763–1775. 24525439 10.1183/09031936.00193413 PMC4040181

[7] Laurence YV, Costs to health services and the patient of treating tuberculosis: a systematic literature review. Pharmacoeconomics. 2015;33(9):939–955. 25939501 10.1007/s40273-015-0279-6 PMC4559093

[8] Hargreaves JR, The social determinants of tuberculosis: from evidence to action. Am J Public Health. 2011;101(4):654–662. 21330583 10.2105/AJPH.2010.199505 PMC3052350

[9] World Health Organization. The End TB Strategy. Geneva, Switzerland: WHO, 2013.

[10] World Health Organization. Tuberculosis patient cost surveys: a handbook. Geneva, Switzerland: WHO, 2017.

[11] World Health Organization. National surveys of costs faced by tuberculosis patients and their households 2015–2021. Geneva, Switzerland: WHO, 2022.

[12] Chittamany P, First national tuberculosis patient cost survey in Lao People’s Democratic Republic: assessment of the financial burden faced by TB-affected households and the comparisons by drug-resistance and HIV status. PLoS One. 2020;15(11):e0241862. 33180777 10.1371/journal.pone.0241862 PMC7660466

[13] Elsayed H, The prevalence of undernutrition and associated risk factors in people with tuberculosis in Lao PDR. SSRN 2024; 10.2139/ssrn.4845382 [Preprint] PMC12180643 40540510

[14] World Health Organization. Protocol for survey to determine direct and indirect costs due to TB and to estimate proportion of TB-affected households experiencing catastrophic total costs due to TB. Geneva, Switzerland: WHO, 2015.

[15] Yamanaka T, Comparing disease specific catastrophic cost estimates using longitudinal and cross-sectional designs: the example of tuberculosis. Soc Sci Med. 2024;344:116631. 38308959 10.1016/j.socscimed.2024.116631

[16] Yamanaka T, Costs incurred by people with co-morbid tuberculosis and diabetes and their households in the Philippines. PLoS One. 2024;19(1):e0297342. 38271328 10.1371/journal.pone.0297342 PMC10810501

[17] Drummond M. Methods for the economic evaluation of health care programmes. 3^rd^ ed. Oxford, UK: Oxford University Press, 2005.

[18] Miyoshi M, Nutritional status of children in rural Lao PDR: who are the most vulnerable? Eur J Clin Nutr. 2005;59(7):887–890. 15915154 10.1038/sj.ejcn.1602160

[19] World Bank. Multi-sector convergence approach to reducing malnutrition in Lao PDR. Washington, DC, USA: World Bank, 2022.

[20] Morishita F, Mitigating financial burden of tuberculosis through active case finding targeting household and neighbourhood contacts in Cambodia. PLoS One. 2016;11(9):e0162796. 27611908 10.1371/journal.pone.0162796 PMC5017748

[21] Vo LNQ, Socio-protective effects of active case finding on catastrophic costs from tuberculosis in Ho Chi Minh City, Viet Nam: a longitudinal patient cost survey. BMC Health Serv Res. 2021;21(1):1051. 34610841 10.1186/s12913-021-06984-2 PMC8493691

[22] Iem V, Pooled testing of sputum with Xpert MTB/RIF and Xpert Ultra during tuberculosis active case-finding campaigns in Lao People's Democratic Republic. BMJ Glob Health. 2022;7(2):e007592. 10.1136/bmjgh-2021-007592 PMC8845188 35165095

[23] Florentino JL, Expansion of social protection is necessary towards zero catastrophic costs due to TB: the first national TB patient cost survey in the Philippines. PLoS One. 2022;17(2):e0264689. 35226705 10.1371/journal.pone.0264689 PMC8884492

[24] Hoa NB, Nhung NV. National tuberculosis patients cost survey: research findings lead to change in policy and practice, Viet Nam. Public Health Action. 2019;9(2):50–52. 31417852 10.5588/pha.18.0082 PMC6645445

[25] Li A, Prevalence and risk factors of malnutrition in patients with pulmonary tuberculosis: a systematic review and meta-analysis. Front Med (Lausanne). 2023;10:1173619. 37636566 10.3389/fmed.2023.1173619 PMC10448260

