# Supplementary material for: Does nutritional support contribute to mitigating the financial burden faced by TB-affected households?
Source: IJTLD Open. 2025 May 12;2(5):260–8. doi: 10.5588/ijtldopen.25.0079 (PMC12068456; doi:10.5588/ijtldopen.25.0079)

http://dx.doi.org/10.5588/ijtldopen.25.0079

Does nutritional support contribute to mitigating the financial burden faced by TB-affected households?

Supplementary Data

Text S1 Additional information on study design

Study sites: Mittaphap hospital and Setthathirath hospital (Vientiane capital), Khammuan provincial hospital (Khammuan province), Luanprabang provincial hospital (Luanprabang province), Savannakhet provincial hospital (Savannakhet province) and Champasack provincial hospital (Champasack province)

Inclusion criteria: 1) participants (including children) who are newly diagnosed as drug-susceptible TB (DS-TB); 2) consent to the study participation within seven days after TB diagnosis; 3) including new, relapse and retreatment TB cases. Data collection was conducted for four times over TB treatment.

Cost components: Direct medical costs (including consultation, drugs, diagnostic tests and hospitalization), direct non-medical costs (including transportation, food and nutritional supplements, and accommodation), and indirect costs (income losses).

Indirect cost estimates: Indirect cost was measured using a difference in self-reported household income at each time point of data collection to estimate income changes during a TB episode. For TB-affected households reporting zero income before having TB, annual household income was imputed using a regression model based on household assets information**.**

Sample size: A sample size of 156 TB patients in each group has 80% power with 0.08 significant level to detect a minimum size of 12.6% reduction in the proportion of patients who incur catastrophic costs. We assumed 90% of nutritional supplement costs would be decreased in intervention group when proper nutritional counselling and feedings were provided, and in this case, the proportion of catastrophic costs will decline from 62.5% to 49.9% based on the result of Lao TB patient cost survey (Chittamany et al Plos One 2020).

Text S2 Details of nutritional interventions provided in this study

Nutrition management/feeding:

The nutritional interventions of this study were provided by trained dieticians who were hired by national nutrition programme of Lao Ministry of Health. Study participants diagnosed with TB with very severe malnutrition (BMI≤16.5 in adults or MUAC<11.5 cm in children) had medical complication and/or no/poor appetite were provided with F-75 and F-100 based on national guidelines on Integrated Management of Acute Malnutrition (IMAM). A Ready-to-Use Therapeutic Food (RUTF) such as Plumpy Nuts was provided for participants who had a good appetite with severe malnutrition (BMI≤16.5 in adults or MUAC<11.5 cm in children) until they recovered to the level of BMI≥16.5 in adults or MUAC≥11.5 cm and <12.5 cm in children. The necessary amount was calculated based on daily calorie intake per patient's weight - 40kcal/kg/day. Given 1 package of RUTF (Plumpy nut) has 500kcal/package, for a patient with body weight of 60kg, the necessary amount was 4 packages (fraction rounded down) – please also see the table below. We maintained providing the RUTF until the BMI recovered to the level of 16.5<BM≤18.5 in adult or MUAC≥11.5 cm and

<12.5 cm in children. We also ensured dietary allowance of micronutrients as a supplement or provided Plumpy nuts a package per day until recovery of BMI to the level of ≥18.5 in adults or MUAC≥12.5 cm in children. Once reaching normal condition (BMI≥18.5 in adults or MUAC≥12.5 cm in children), we only provided nutritional monitoring and counselling.

Examples of necessary calorie intake and amount of RUTF packages based on body weight

Body Weight	Necessary Calorie per day (40kcal/kg/day)	Amount of RUTF (500kcal/package, fraction rounded down)
**30**	1200 kcal	2 packages
**35**	1400 kcal	3 packages
**40**	1600 kcal	3 packages
**45**	1800 kcal	3 packages
**50**	2000 kcal	4 packages
**55**	2200 kcal	4 packages
**60**	2400 kcal	4 packages

Supplementary Fig S3. Study participant flowchart

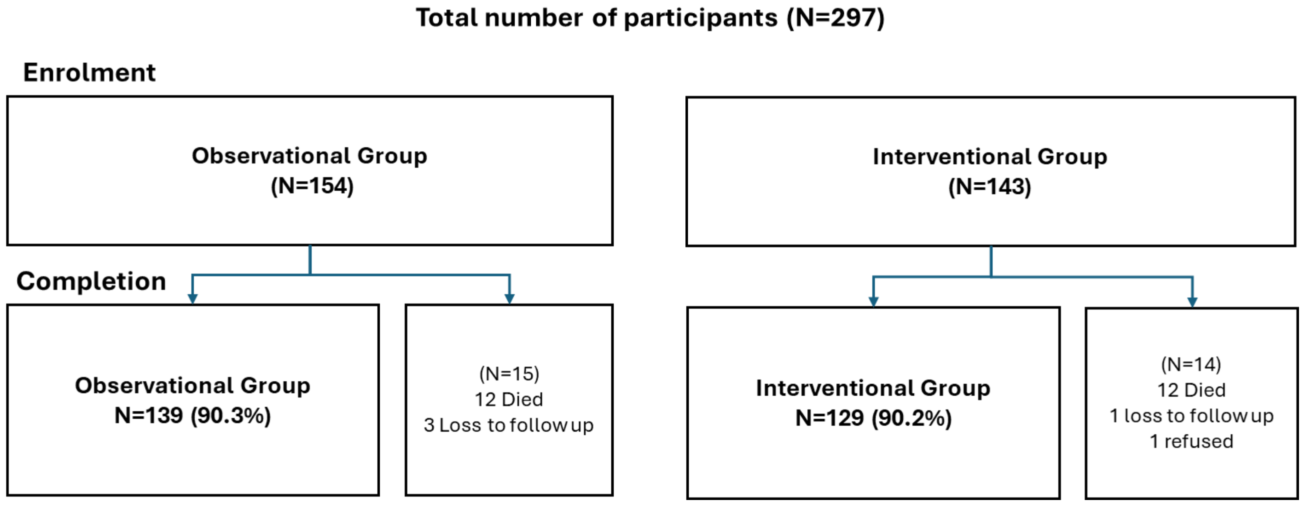

Supplementary Table S4. **Socioeconomic status of study participants, by status of the completion of data collection**

Variables		Complete		Drop out		All enrolment		p-value
		N	(%)	N	(%)	N	(%)	
**Total**		**268**	**90.2%**	**29**	**9.8%**	**297**	**100%**	
**Demographic characteristics**								
Age group	0–14	1	0.4%	0	0.0%	1	0.3%	0.865
	15–24	24	9.0%	3	10.3%	27	9.1%	
	25–34	50	18.7%	3	10.3%	53	17.8%	
	35–44	37	13.8%	3	10.3%	40	13.5%	
	45–54	51	19.0%	5	17.2%	56	18.9%	
	55–64	52	19.4%	7	24.1%	59	19.9%	
	≥65	53	19.8%	8	27.6%	61	20.5%	
Sex	Female	106	39.6%	6	20.7%	112	37.7%	0.074
	Male	162	60.4%	23	79.3%	185	62.3%	
Education level	No education	26	9.7%	4	13.8%	30	10.1%	0.856
	Primary	70	26.1%	6	20.7%	76	25.6%	
	Lower/higher secondary	121	45.1%	13	44.8%	134	45.1%	
	Diploma or higher, vocational, other	51	19.0%	6	20.7%	57	19.2%	
Insurance status	None	95	35.4%	13	44.8%	108	36.4%	0.654
	National Health Insurance (NHI) scheme	148	55.2%	14	48.3%	162	54.5%	
	Community-Based Health Insurance (CBHI)	2	0.7%	1	3.4%	3	1.0%	
	Health Equity Fund (HEF)	0	0.0%	0	0.0%	0	0.0%	
	Social Security Organization (SSO) for salaried private-sector employees	9	3.4%	0	0.0%	9	3.0%	
	State Authority for Social Security (SASS) for civil servants	10	3.7%	1	3.4%	11	3.7%	
	Private health insurance	3	1.1%	0	0.0%	3	1.0%	
	Other	1	0.4%	0	0.0%	1	0.3%	
Employment status before TB	Unemployed	67	25.0%	8	27.6%	75	25.3%	0.722
	Formal paid work	40	14.9%	6	20.7%	46	15.5%	
	Informal paid work	128	47.8%	13	44.8%	141	47.5%	
	Retired/student/housework/other	33	12.3%	2	6.9%	35	11.8%	
Household size	≥5	130	48.5%	19	65.5%	149	50.2%	0.122
	<5	138	51.5%	10	34.5%	148	49.8%	
Primary income earner	No	162	60.4%	18	62.1%	180	60.6%	0.925
	Yes	91	34.0%	9	31.0%	100	33.7%	
	Equal contributor	15	5.6%	2	6.9%	17	5.7%	
**Clinical characteristics**								
TB type	Pulmonary, bacteriologically confirmed	189	70.5%	23	79.3%	212	71.4%	0.438
	Pulmonary, bacteriologically unconfirmed	65	24.3%	4	13.8%	69	23.2%	
	Extrapulmonary	14	5.2%	2	6.9%	16	5.4%	
Treatment history	New	254	94.8%	27	93.1%	281	94.6%	0.375
	Relapse	12	4.5%	1	3.4%	13	4.4%	
	Retreatment	2	0.7%	1	3.4%	3	1.0%	
HIV status	HIV positive	32	11.9%	9	31.0%	41	13.8%	0.018
	HIV negative	235	87.7%	20	69.0%	255	85.9%	
	Status unknown	1	0.4%	0	0.0%	1	0.3%	
Diagnostic delay*	(>4weeks)	133	49.6%	13	44.8%	146	49.2%	0.768
Body mass index at TB diagnosis	<18.5	101	37.7%	16	55.2%	117	39.4%	0.103
	≥18.5	167	62.3%	13	44.8%	180	60.6%	
**Financial status**		**Mean**	**95%CI**	**Mean**	**95%CI**	**Mean**	**95%CI**	
Self-reported monthly household	Before onset of TB symptoms	366	(297–434)	493	(81–904)	378	(305–451)	0.308
	At the time of TB diagnosis	317	(251–384)	252	(171–333)	311	(250–371)	0.532

Income (in US$)								

Supplementary Fig S5. Coping mechanisms (a) and social consequences (b) faced by TB-affected households, by TB treatment phase

A high incidence of job loss was observed before TB diagnosis (19.8%) and during intensive phase (11.9%) which may have resulted in dissaving and taking loans to cope with financial burden due to TB during these periods.

(a) coping mechanisms

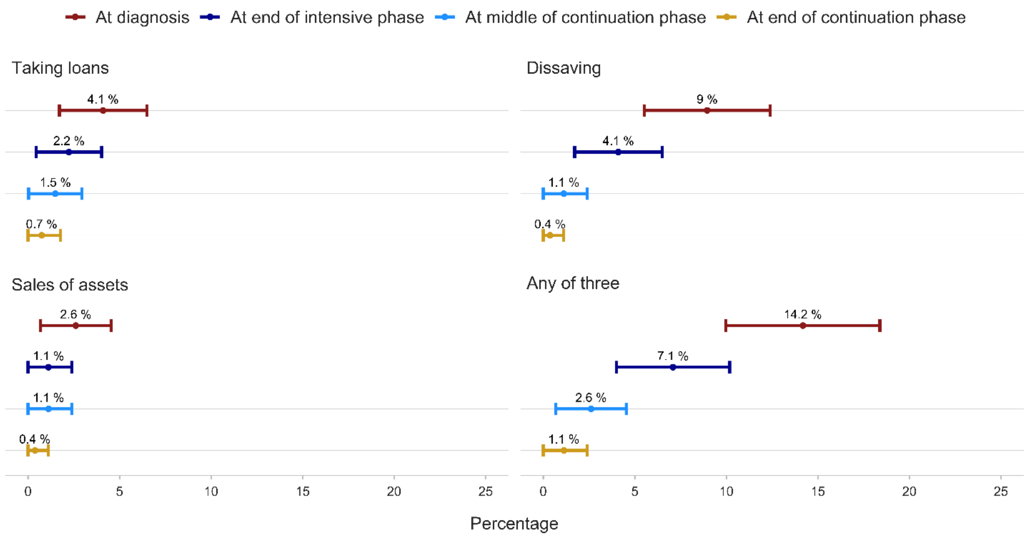

(b) social consequences

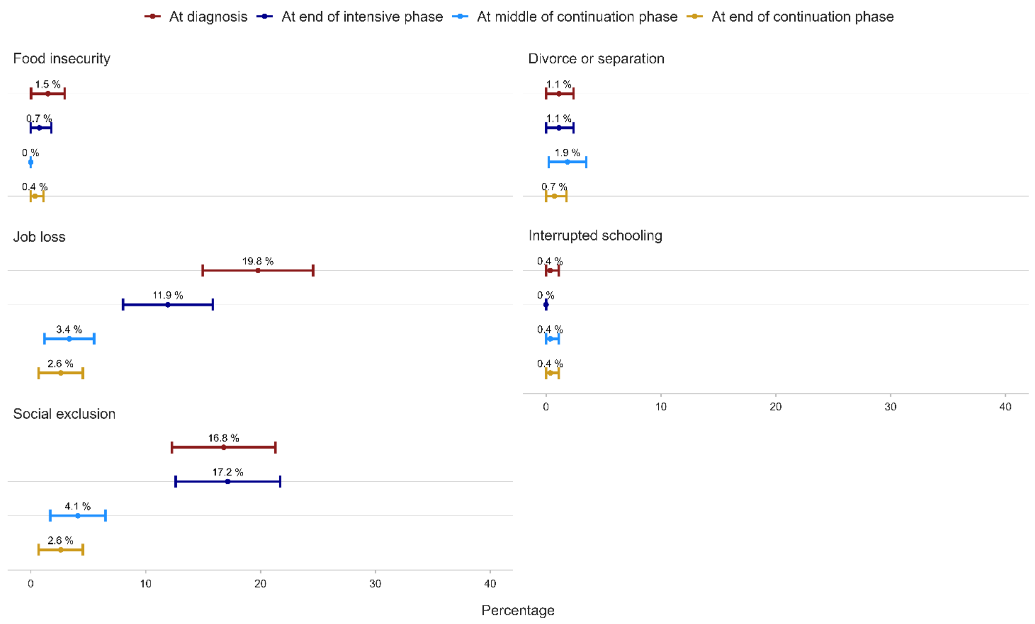

Supplement: Supplementary file 1 [file ijtldopen25-0079_supplementarydata1.pdf]
